# Local and systemic immunomodulatory mechanisms triggered by Human Papillomavirus transformed cells: a potential role for G-CSF and neutrophils

**DOI:** 10.1038/s41598-017-09079-3

**Published:** 2017-08-21

**Authors:** Karla Lucia Fernandez Alvarez, Mariana Beldi, Fabiane Sarmanho, Renata Ariza Marques Rossetti, Caio Raony Farina Silveira, Giana Rabello Mota, Maria Antonieta Andreoli, Eliana Dias de Carvalho Caruso, Marcia Ferreira Kamillos, Ana Marta Souza, Haydee Mastrocalla, Maria Alejandra Clavijo-Salomon, José Alexandre Marzagão Barbuto, Noely Paula Lorenzi, Adhemar Longatto-Filho, Edmund Baracat, Rossana Verónica Mendoza Lopez, Luisa Lina Villa, Maricy Tacla, Ana Paula Lepique

**Affiliations:** 1 Department of Immunology, Institute of Biomedical Sciences, Universidade de São Paulo, Av. Prof. Lineu Prestes, 1730, Ed. Biomédicas IV, 05508-900 São Paulo, SP Brazil; 2 Department of Gynecologic Clinic, School of Medicine, Universidade de São Paulo; Clinics Hospital at the São Paulo University, R. Dr. Enéas de Carvalho aguiar, 255, 5th floor, 05403-000 São Paulo, SP Brazil; 3 Department of Radiology and Oncology, Faculdade de Medicina da Universidade de São Paulo, LIM-24. R. Dr. Ovídio Pires de Campos, 255, Radiology Building, 05403-000 São Paulo, SP Brazil; 4 Center for Translational Research in Oncology, Instituto do Câncer do Estado de São Paulo, Av. Dr. Arnaldo, 251, 8th floor, 01246-000 São Paulo, SP Brazil; 5 Laboratory of Medical Investigation, School of Medicine, University of São Paulo, Av. Dr. Arnaldo, 455, office 1159, 01246-903 São Paulo, SP Brazil; 6 Molecular Oncology Research Center, Barretos Cancer Hospital, R. Antenor Duarte Vilela, 1331, Barretos, 14784-400 São Paulo, SP Brazil; 7 Life and Health Sciences Research Institute (ICVS), School of Health Sciences, University of Minho, R. da Universidade and ICVS/3B’s - PT Government Associated Laboratory, 4704-553 Braga/Guimarães, Portugal; 8 Hospital AC Camargo, International Research Center, R. Taguá 440, 01508-010 São Paulo, Brazil

## Abstract

Cervical cancer is the last stage of a series of molecular and cellular alterations initiated with Human Papillomavirus (HPV) infection. The process involves immune responses and evasion mechanisms, which culminates with tolerance toward tumor antigens. Our objective was to understand local and systemic changes in the interactions between HPV associated cervical lesions and the immune system as lesions progress to cancer. Locally, we observed higher cervical leukocyte infiltrate, reflected by the increase in the frequency of T lymphocytes, neutrophils and M2 macrophages, in cancer patients. We observed a strong negative correlation between the frequency of neutrophils and T cells in precursor and cancer samples, but not cervicitis. In 3D tumor cell cultures, neutrophils inhibited T cell activity, displayed longer viability and longer CD16 expression half-life than neat neutrophil cultures. Systemically, we observed higher plasma G-CSF concentration, higher frequency of immature low density neutrophils, and tolerogenic monocyte derived dendritic cells, MoDCs, also in cancer patients. Interestingly, there was a negative correlation between T cell activation by MoDCs and G-CSF concentration in the plasma. Our results indicate that neutrophils and G-CSF may be part of the immune escape mechanisms triggered by cervical cancer cells, locally and systemically, respectively.

## Introduction

Cervical cancer is the fourth most common cancer in women worldwide and, according to the World Health Organization (WHO) responsible for 7.5% of women’s death by cancer. HPV infection is the main etiological factor for cervical cancer development^1^. After HPV infection, the natural history of cervical cancer is long and, in general, develops through low and high grade cervical intraepithelial neoplasia (CIN) and finally cancer. The lower female genital tract has a diffuse lymphoid tissue, prepared to respond to potential injuries and infections^2^. The cervix is anatomically more susceptible to HPV infection, which promotes immune responses from the infected cells and innate immune cells, as well as adaptive immune responses. It is well known that CD4Th1 cells respond to viral antigens secreting IFNγ, TNFα and IL-2 and that CD8 T cells can promote wart and lesion regression in humans^3–5^. Moreover, immunosuppressed patients are at higher risk of infection by HPV and lesion development^6^, indicating that the immune system is important in preventing lesion progression and promoting infection clearance. Although immune responses against HPV may be robust, there are regulatory and evasion mechanisms that can inhibit T cell responses. For instance, in cancer patients, regulatory T cells play an important role in the suppression of anti-tumor immune responses^7, 8^. There is also a positive correlation between macrophage infiltration and lesion grade in the infected cervical tissues^9–11^. M2 or alternatively activated macrophages have been shown to promote angiogenesis, resistance to chemotherapy and suppression of T cell responses in cervical cancer patients^12–14^. Tumors also cause systemic effects on the immune system. Recently, leukocytosis has been shown to be a poor prognostic factor for patients with recurrent cervical cancer, as well as a marker for resistance to chemotherapy^15^. The circulating neutrophil/lymphocyte ratio (NLR) has been used as a cancer prognostic marker. In patients with cervical cancer, high NLR indicates poor prognostic and resistance to chemo and radiotherapy^16^.

However, the interaction between the various leukocyte populations infiltrating cervical lesions and the systemic effects of these lesions on the immune system are not so well characterized, mainly if we look at precursor lesions compared to cancer. The objective of this study was to characterize the inflammatory infiltrate in low and high grade CIN and cervical cancer, compared to cervicitis, a non-neoplasic cervical inflammation, which can be caused by different etiological factors. We have also investigated cervical lesions systemic effects, such as G-CSF plasma concentration, frequency of circulating immature neutrophils and different populations of dendritic cells and antigen presentation potential of monocyte derived dendritic cells (MoDC), since these are factors commonly associated with cancer tolerance mechanisms in both cervical cancer patients and other cancer patients^15, 16^.

## Results

### Increase in the frequency of T lymphocytes, neutrophils and M2 macrophages in cancer samples compared to precursor cervical lesions

Although HPV infection triggers immune responses, a fraction of the infected women are persistently infected and eventually develop cervical cancer. The importance of the immune system in clearing the infection is clear^6^. However, how different leukocyte populations interact with each other and the microenvironment throughout lesion progression is not so well understood. Therefore, we sought to characterize the global leukocyte infiltrate in samples of cervicitis, CIN 1, 2 and 3, and invasive cervical cancer (ICC) by flow cytometry, which allowed us to study several leukocyte populations simultaneously. Our patients were recruited through the Gynecology service at Hospital das Clínicas in São Paulo, to which they were referred after being diagnosed with positive cytology for HPV associated cervical lesions in their local medical services.

**Figure 1:**
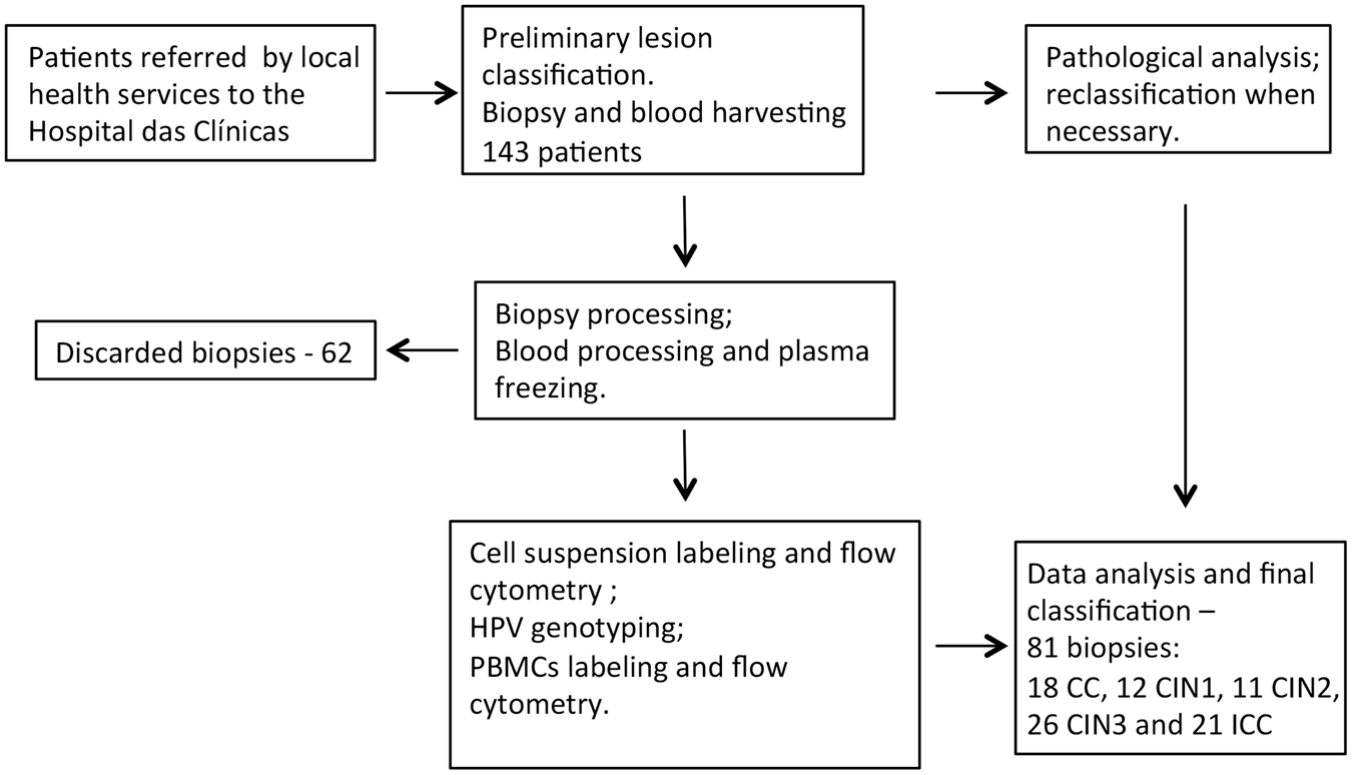
Organogram of cohort enrolment. Patients with cytology indicative of HPV associated lesion were referred to Hospital das Clínicas. A total of 143 patients were enrolled with colposcopy suspicious of cervical lesion. Biopsies and peripheral blood were processed and if we obtained enough cells from biopsies, these cells were labeled with antibodies to continue the study, if not, only the blood sample was saved. From the 62 discarded biopsies, 54 did not have enough cells; the others were discarded because there was not enough patient’s clinical data. Several of the harvested and analyzed samples were reclassified according to the histopathological classification determined by the Pathology service at Hospital das Clínicas. This meant that flow cytometry analyses were performed as blind assays regarding lesion grade, which avoided any kind of bias by the student conducting the experiments. In the end of the enrolment period, we had 81 analyzed biopsies distributed in different lesion grades as indicated in the figure.

Table 1 shows this study’s demographic data and Fig. 1 shows an organogram of patients’ enrolment and number of cervical samples used. We harvested biopsies from a total of 143 patients. However, we were able to actually analyze and use the data correspondent to 88 biopsies. The main cause for discarding part of the biopsies was the low yield in cell number or viability after biopsy processing. Also, in a few cases, we were unable to retrieve essential clinical data from the patients, and therefore, could not use the results from them.

**Table 1:** Demographic data from patients enrolled in this study.

Characteristic		Cervicitis	CIN1	CIN2	CIN3	Invasive Ca
Number of analyzed biopsies^a^		n = 24	n = 14	n = 25	n = 46	n = 34
Number of analyzed blood samples^a,b^		8	6	17	14	17
Age (yrs)	median	38	28	36	33	45^d^
	range	24–81	19–67	23–59	21–57	24–75
Parity	mean	2	2	2	2	3
	range	0–5	0–4	0–4	0–5	0–11
Sexual debut (yrs)	median	17	18	19	16	16
	range	15–20	14–23	14–23	12–24	14–18
Menopause	percentage	0	10	18	4	28
Smokers	percentage	28	27	18	29	22
HPV infection	percentage	78	92	86	95	95
High risk HPV infection	percentage	65	83	86	92	95
Multiple HPV infection^c^	percentage	30	42	36	28	9
Tumor staging (FIGO)	IA1 (%)					13
	IB1 (%)					13
	IB2 (%)					7
	IIA1 (%)					7
	IIB (%)					33
	III (%)					7
	IIIB (%)					7
	IV (%)					7
	IVA (%)					7

^a^In each experimental group there was a varied proportion of patients from whom we were able to analyzed both the cervical biopsy and blood samples; ^b^blood samples include PBMCs, neutrophils and plasma, not all samples were analyzed for all different parameters; ^c^the multiple HPV infection group is a percentage of the total analyzed samples, and is included in the HPV positive infection group; ^d^there was a significant positive correlation between age and lesion grade. Patients with cancer were significantly older than patients with other lesions (tested by one-way ANOVA, p = 0.03). Invasive Ca – invasive carcinoma.

As expected, we observed that ICC patients were significantly older than patients with precursor cervical lesions or cervicitis^17^. Other factors as parity, menopause and smoking habits did not vary significantly among the different groups, probably due to the number of subjects in each group. HPV DNA was detected in samples from all the groups, although in different frequencies. As expected, a positive correlation between frequency of high oncogenic risk HPV infection and lesion grade was observed in our cohort (Table 1). However, the frequency of HPV infection in cervicitis patients (78%) was higher than expected. This was probably due to our cohort design that recruited patients with indication of altered cytology. It is likely that in the average six months period, between cytology diagnostic and visit to the Gynecology service at Hospital das Clínicas, part of the patients spontaneously eliminated their lesions, though not the HPV infection.

**Figure 2:**
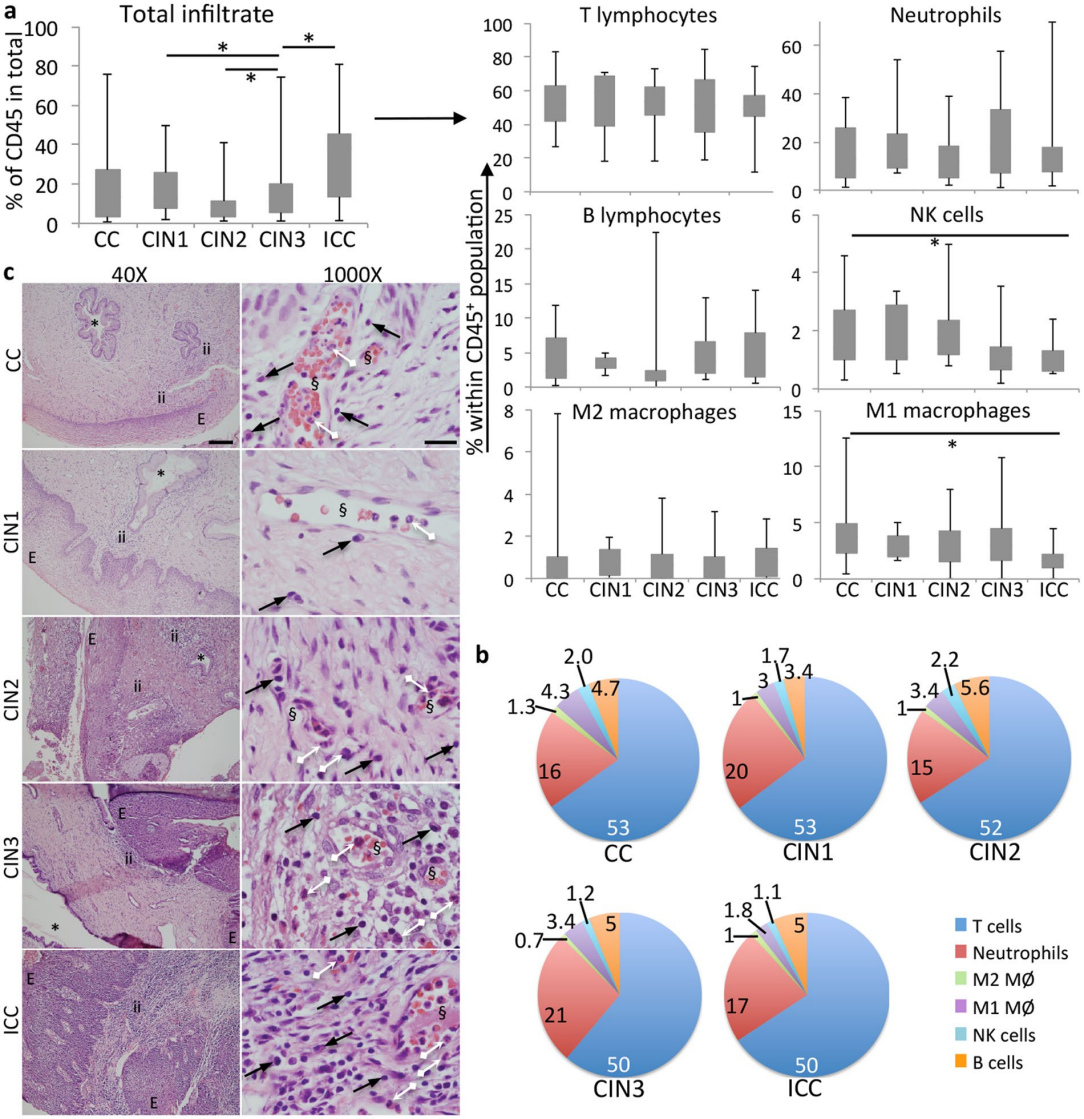
Leukocyte populations infiltrating cervical lesions. (**a**) Flow cytometry analyses of cervical single cell suspensions labeled with antibodies against cell surface markers. Total infiltrate corresponds to all CD45^+^ cells (box plot left side), all other box-plots are cells gated in the CD45^+^ population. Phenotype of each population is specified in Supplemental Material 1. Number of patients in each group is specified in Fig. 1. (**b**) Frequency of different cell populations per patient group. The values correspond the average frequency of each population; standard deviation values were omitted in the sake of space. Populations’ frequencies were compared by one-way ANOVA, ^*^indicates significant differences in cells frequency among groups. (**c**) Histology of cervical tissues stained with HE. Image magnification is indicated, as well as lesion grade. E – epithelial compartment, ii – inflammatory infiltrate, * - glandular tissue, § - blood vessels, black arrows point to mononuclear cells, white arrows point to granulocytes. CC – cervicitis, CIN 1, 2, 3 – Cervical Intraepithelial Neoplasia grades 1, 2, 3, ICC – invasive cervical cancer. Scale bars indicate 250 μm and 50 μm in 40X and 1000X magnification micrographs (upper panels), respectively.

Single cell suspensions obtained from colposcopy driven biopsies, with 90% or more viable cells, were labeled with antibodies against several leukocyte cell surface markers and analyzed by flow cytometry (Supplementary Information 1a,b). As can be observed in Fig. 2, there was some spread in the leukocyte frequency of our samples. In order to facilitate data analyzes, we opted to show the frequency of specific leukocyte populations within the gate that defined the CD45^+^ pan-leukocyte one. We observed that the global leukocyte infiltration (CD45^+^ cells) was significantly higher in ICC than in CIN lesions and cervicitis (Fig. 2a,c). This increase was due to the increase in T lymphocytes (SSC^low^CD45^+^CD3^+^CD19^−^), neutrophils (SSC^high^CD45^+^CD16^+^HLA-II^−^CD11b^+^CD86^−^) and M2 macrophages (CD45^+^CD3^−^CD11b^+^CD16^+^HLA-DR^+^CD64^+^CD86^+^CD206^+^, population defined by positive CD206 expression^18^) frequencies. One can notice that the frequency of these populations was unchanged within the CD45^+^ population, indicating they varied accordingly to the parental CD45^+^ population, therefore, increasing in cancer samples. On the other hand, we observed that within the CD45^+^ population, the frequency of NK (SSC^low^CD3^−^CD16^+^) cells and M1 macrophages (CD45^+^CD3^−^CD11b^+^CD16^+^HLA-DR^+^CD64^+^CD86^+^CD206^−^) was significantly lower in cancer samples than in precursor lesions. Together, M1 macrophages and NK cells accounted for 6.3, 5.2, 5.6, 4.6 and 2.9 percent of the total leukocyte population in cervicitis, CIN1, 2, and 3 and cervical cancer biopsies, respectively (Fig. 2c). Therefore, in average and in comparison to the other leukocyte populations infiltrating the cervical lesions, there was a relative decrease of 2.17 fold in M1 and NK combined populations in cancer when compared to cervicitis and 1.77 fold in relation to CIN lesions. These results suggest a change in the cervical microenvironment as lesions progression from low and high grade intraepithelial neoplasia to cancer.

We have detected infiltrating B lymphocytes in a percentage, but not all of the cervical lesions (Fig. 2a,b). We also investigated the T cell subpopulations CD4 and CD8 (SSC^low^CD45^+^CD19^−^CD3^+^CD4^+^CD8^−^ and SSC^low^CD45^+^CD19^−^CD3^+^CD4^−^CD8^+^, respectively) but found that they varied according to the T cell total population (data not shown). Finally, we did not find a Lin^−^CD11b^+^CD33^+^ population in our biopsies, which would correspond to myeloid derived suppressor cells (MDSC)^19^, nor did neutrophils express CD33 (Lin corresponds to lineage markers, used to gate out lymphocytes, granulocytes and monocytes from the cytometry analysis).

Our flow cytometry data was confirmed by histopathological analyzes of hematoxylin/eosin (HE) stained cervical biopsies. We could observe monocytic (black arrows, pointing to lymphocytes and macrophages) and polymorphonuclear cells infiltrating in the biopsies (white arrows) (Fig. 2c). Although not by any means quantitative, there was a good correlation between the flow cytometry and histology data, regarding the total inflammatory infiltration (Fig. 2c,ii) in the cervical lesions, as well the infiltration by monocytic and polymorphonuclear cells (Fig. 2c). Importantly, in very few biopsies we found extensive hemorrhagic areas, so that the leukocytes detected by flow cytometry were the result of active recruitment, rather than blood vessels disruption.

Infection by other pathogens may have a role in leukocyte recruitment to the cervix. Data from patients’ files indicated, in a few cases, other microbial infections besides HPV. We found indication of infection by *Gardnerella* or cocci in a total of 10 patients, in the cervicitis and CIN1 groups, and Chlamydia infection in a CIN3 sample. We have compared the leukocyte infiltrate in biopsies from patients with and without indication of bacterial infection and found no significant differences (data not shown).

### Negative correlation between T cells and neutrophils differentiated CINs and cancer from cervicitis

**Figure 3:**
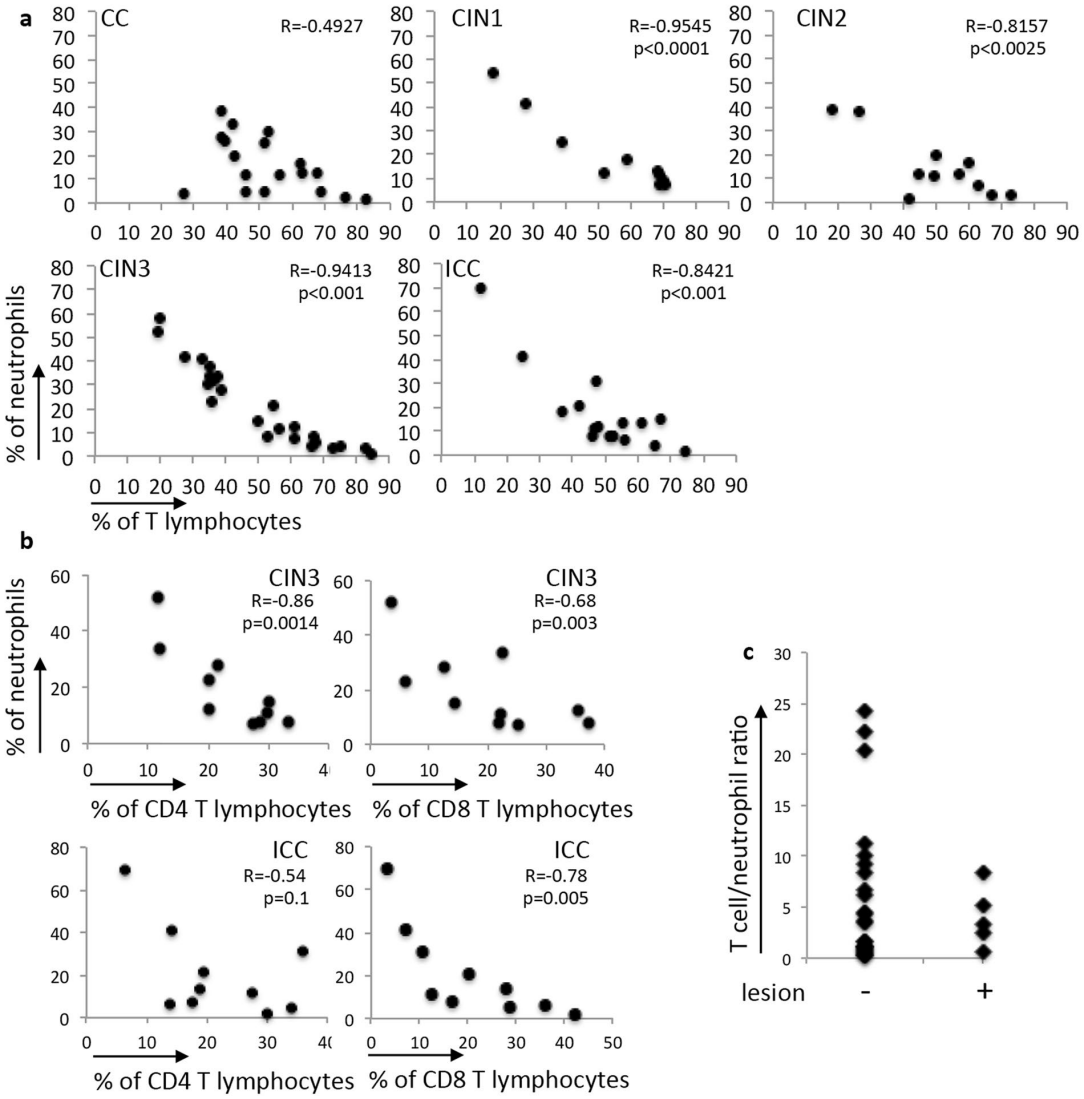
Negative correlation between neutrophils and T cells in CIN and ICC. (**a**) Frequencies of T cells and neutrophils in each cohort group were compared by Pearson correlation. Number of samples per group is described in Fig. 1. (**b**) Comparison between CD4 and CD8 and neutrophil frequencies in CIN3 and ICC samples by Pearson correlation. There were 9 CIN3 biopsies and 11 ICC biopsies in these analyses. The graphs indicate the R values and p values (significance) for each correlation. (**c**) T cell/neutrophil frequency ratio in patients with CIN1, 2 and 3 free of disease (−) or with persistent or recurrent disease (+) after excision procedure. Follow up period varies between 1 and 3 years.

T lymphocytes and neutrophils were the most abundant cells in the leukocyte infiltrate of the cervical lesions. Their frequencies varied among patients, ranging from 11.8 to 82.8 percent of CD45^+^ population, for T cells, and 1 to 69 percent for neutrophils. In cervical cancer patients, the circulating NLR has been used as prognostic marker^16^. We asked whether there was any correlation between neutrophils and T cells in the cervix. As can be observed in Fig. 3a, there was a strong and significant negative correlation between neutrophils and T cells in all CIN lesions and ICC, but not in cervicitis. To further understand the interaction between neutrophils and T cells, we repeated this analyzes separating T cells in CD4 or CD8 subpopulations. Significant negative correlation was observed in CIN3 and in ICC, although the last one only with CD8 T cells (Fig. 3b). We were not able to perform these analyses with CIN1and CIN2 samples (due to the smaller number of cells obtained from these samples, we excluded analyses of CD4 and CD8 subpopulations in approximately 40% of these biopsies, and therefore did not have enough events for correlation analysis). The strong negative correlation between T cells and neutrophils led us to question whether neutrophils could inhibit T cells in the lesion microenvironment^20^. We proceeded to perform *in vitro* assays to verify this possibility. We also raised follow up information from the patients enrolled in the study. Although our follow up period is short, we did obtain data from a few patients and, interestingly, we found that in patients with persistent or recurrent lesions, the T lymphocyte/neutrophil frequency ratio was bellow 8. In other patients free of disease, we found varying T cell/neutrophil ratios (Fig. 3c). Although we need a longer follow up period and larger sample to be able to confirm this data, it seems that recurrent lesions are more likely to be observed in patients with lower T lymphocyte/neutrophil frequency local ratios.

### Neutrophils in cervical biopsies display markers of suppressive neutrophils

**Figure 4:**
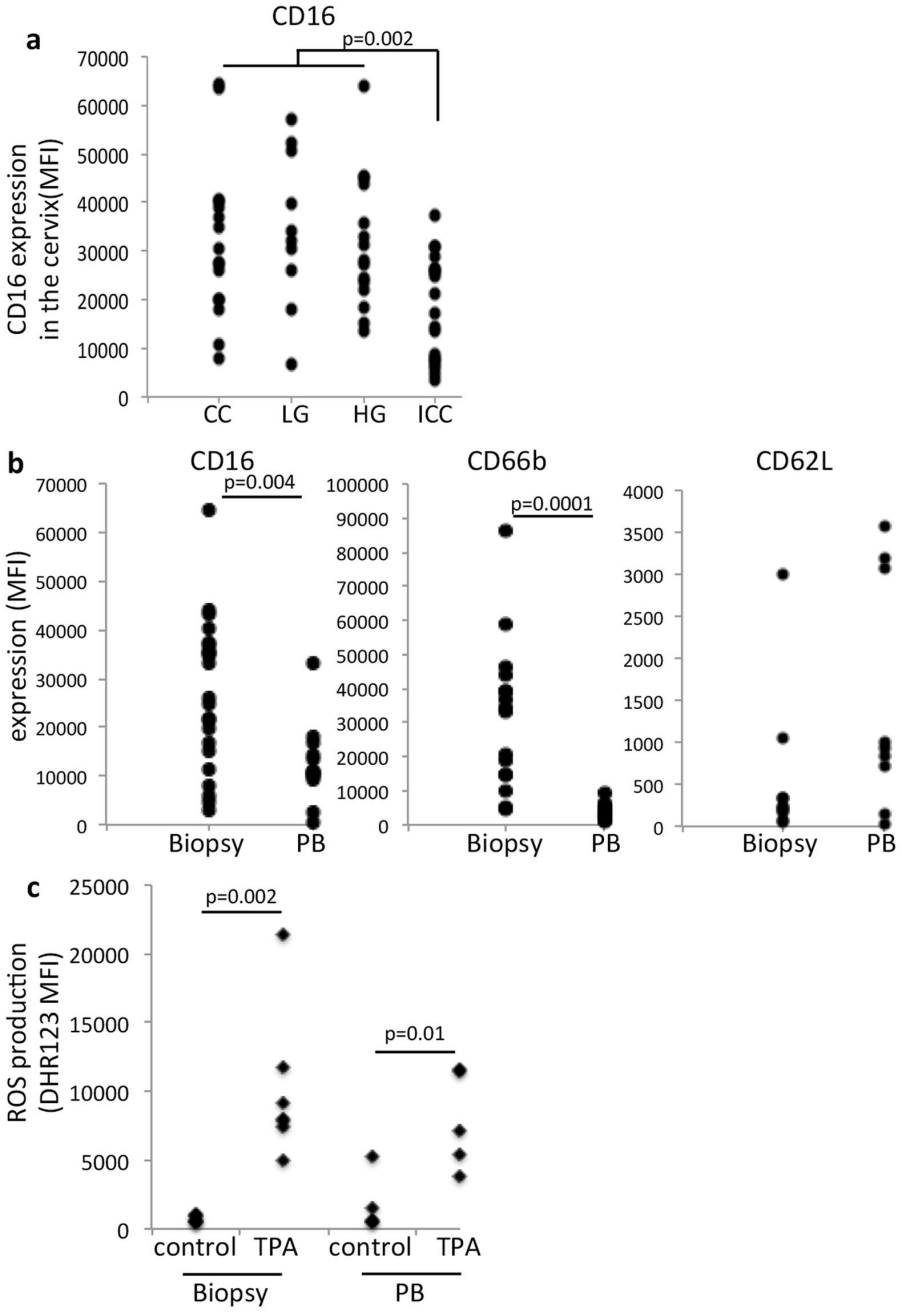
Characteristics of cervical cancer infiltrating neutrophils. (**a**) CD16 expression (median fluorescence intensity, MFI) was evaluated in biopsy neutrophils, after collagenase digestion, labeling with anti-CD33, CD66b, CD11c, CD15, CD11b, CD45, CD62L, and analyzed by flow cytometry. After exclusion of debris and doublets, by SSC-A x FSC-A and FCS-A x FSC-H, cells gated on high SSC and CD45^+^ were evaluated for expression of the indicated makers. The only marker with significant changes among patient groups was CD16. As we had few CIN2 patients to evaluate for this parameter, we joined these patients with CIN3 patients to form a high grade lesion group (HG). CC – cervicitis, LG – low grade lesion group (same as CIN1), ICC – invasive cervical cancer. We evaluated 11 CC patients, 10 LG patients (same as CIN1), 12 HG patients and 15 ICC patients. The indicated p value, was obtained with one-way ANOVA testing. (**b**) Comparison of CD16, CD66b and CD62L expression in biopsy and circulating neutrophils (PB) from ICC patients. Results are displayed as median fluorescence intensity (MFI) and in this case, compared by t-test. The resulting p values are indicated. (**c**) ROS production by biopsy and circulating neutrophils from ICC patients, measured by DHR123 fluorescence. Neutrophils isolated from biopsies or peripheral blood were stimulated with 100 ng/ml TPA for 15 min and then incubated with DHR-123 for 15 min. Cells were then labeled with anti-CD45, CD11b and CD66b. DHR signal, displayed as median fluorescence intensity (MFI), was measured in triple positive cells, after gating out debris and doublets. In this case, our experimental group had 5 patients. Results were tested by t-test between control and treated cells, and the p values are indicated. There was no difference between ROS production in control or treated neutrophils from biopsies compared to circulating cells.

Based on the hypothesis that CIN infiltrating neutrophils could inhibit T cell responses, we first decided to evaluate activation markers in the cells present in the cancer biopsies, compared to circulating neutrophils. Our first observation was that neutrophils from cancer biopsies expressed significantly lower levels of CD16 (FcγR III) than neutrophils from precursor lesions (Fig. 4a). In spite of that, CD16 and CD66b expression levels were significantly higher in neutrophils from biopsies than circulating neutrophils from the same patients (Fig. 4b). Interestingly, biopsy neutrophils expressed low CD62L levels (Fig. 4b). CD16 can signal through Phosphatidylinositol-3 kinase (PI3K) in neutrophils leading to cell activation and increased survival^21^. CD66b, likewise, is a neutrophil activation marker, but may also indicate immunosuppressive activity^22^. Moreover, CD16^high^CD62L^dim^ neutrophils are recognized as suppressive neutrophils, with low ROS production capacity and suppression of T cell activity^23, 24^. Therefore, our results added to our hypothesis that neutrophils in cervical cancer could be suppressive neutrophils.

To test the activity of cervical cancer neutrophils, we sorted and activated neutrophils from blood or ICC biopsies (number of cells recovered from CIN did not allow us to perform this type of assay with other lesions) and stimulated with 12-O-Tetradecanoylphorbol-13-acetate (TPA) *ex vivo*. Neutrophils from ICC were able to generate similar concentrations of reactive oxygen species (ROS) as circulating neutrophils from the same patients upon stimulation (Fig. 4c). Therefore, neutrophils from cancer biopsies were alive and functional, able to activate NADPH oxidase upon stimulation.

**Figure 5:**
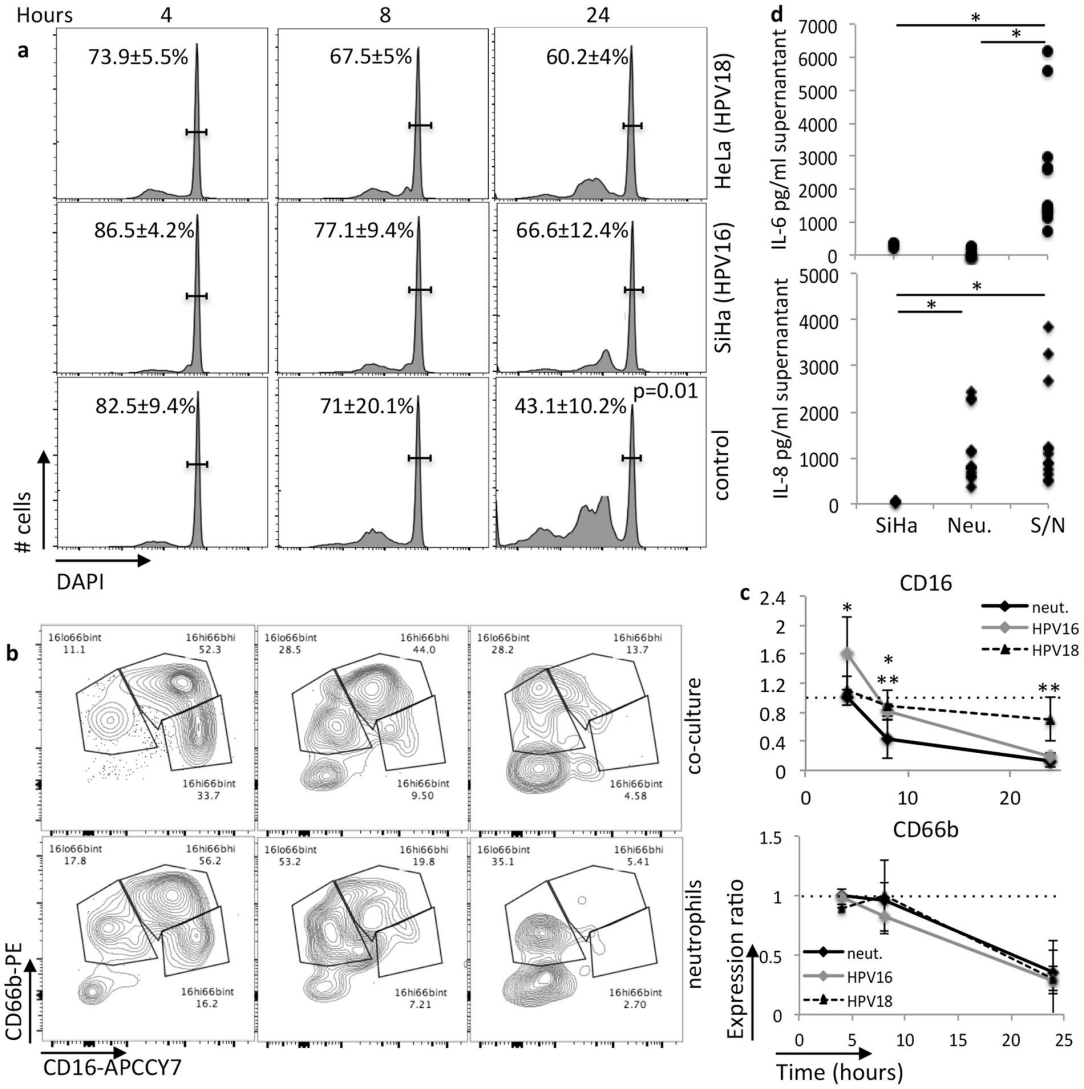
Cross-talk between cervical cancer derived cells and neutrophils. Neutrophils in co-culture with SiHa spheroids (co-culture) or in pure cultures (neutrophils) were evaluated at the indicated periods of time for: (**a**) viability, (**b**) CD66b and CD16 expression, and (**c**) cytokine production (only at 24 hours of culture). At each time point, cells and supernatants were harvested, cells were labeled with anti-CD45, CD66b, CD16, fixed and incubated with 10 μg/ml DAPI for 30 min before flow cytometry analysis. A. DAPI fluorescence was measured in the CD45^+^ population. The gated cells in the histograms indicate DNA content corresponded to whole cells, events to the left, correspond to sub-G1 dead cells. Significant differences were found only at 24 hours of culture (p value indicated). Still within the CD45^+^ population, we evaluated frequency (**b**) and expression ratio (**c**), depicted as expression of CD66b and CD16 in co-cultures/expression in pure neutrophil cultures. The frequency plots are representative of one experiment, and the kinetic in the right side graphs are the average ratios obtained from all experiments. A total of 5 different blood samples were used in these experiments. The U-test Mann Whitney tested differences in CD16 and CD66b expression ratios through time; ^*^indicates significant differences in CD16 expression between experimental conditions; dotted lines indicate ratio 1 for reference. (**d**) Cytokine production. Supernatants from 24 hours cultures were tested for cytokine production using the Human Inflammatory CBA kit, according to the manufacture’s instructions. Although this kit has antibodies for several cytokines, only IL-6 and IL-8 were secreted in concentrations above cut off values. SiHa – tumor cells spheroid supernatant, Neu. – neutrophils supernatant, S/N – SiHa spheroids and neutrophil co-cultures. We tested the results using one-way ANOVA, ^*^indicates significant differences between experimental groups.

We decided to further investigate the effect of tumor cells on neutrophils by using an experimental model with SiHa and HeLa tumor spheroids (HPV16 squamous cell carcinoma and HPV18 adenocarcinoma derived cell lines, respectively. These are the most common HPV types in cervical cancer patients)^25^. Circulating neutrophils from healthy donors were incubated alone or with SiHa or HeLa spheroids for 4, 8 and 24 hours. We observed, as shown in Fig. 5a, that after 24 hours of culture, there was a significant higher percentage of live neutrophils in co-cultures, than in neat neutrophil cultures (1.56 and 1.4 fold more live neutrophils in co-cultures, respectively). We also observed that CD16 expression half-life was significantly shorter in neat neutrophil cultures than in neutrophils in co-culture with SiHa or HeLa cells (Fig. 5b,c), which could be the cause for the extended half-life of these cells in co-culture with tumor cells^21^. CD66b expression had no significant alteration between the two experimental groups (Fig. 5b,c). Interestingly, in the cervical biopsies, we also did not observe significant alteration of CD66b expression among the different lesion grades, indicating that this molecule was upregulated upon recruitment, but not modulated by the cervical cells.

Tissue processing could activate neutrophils. Therefore, we processed circulating neutrophils in the exact same way as cells from biopsies and observed that several surface markers were up regulated: CD11c, CD62L, CD45, CD11b. CD66b was marginally activated by manipulation (1.3 fold), and CD16 expression was not altered (data not shown), again indicating that CD16 expression was modulated by tumor cells.

Finally, we also observed a 7.7 fold increase in IL-6 secretion in SiHa and neutrophils co-cultures compared with SiHa cell cultures, after 24 hours incubation. IL-8 was expressed by neutrophils alone and there was no significant increase in its secretion by co-culture with SiHa (Fig. 5d). We also investigated expression of IL-1β, IL-10, IL-12 and TNFα, and found no expression of these cytokines in these cultures.

### Neutrophils controlled T cell activity

**Figure 6:**
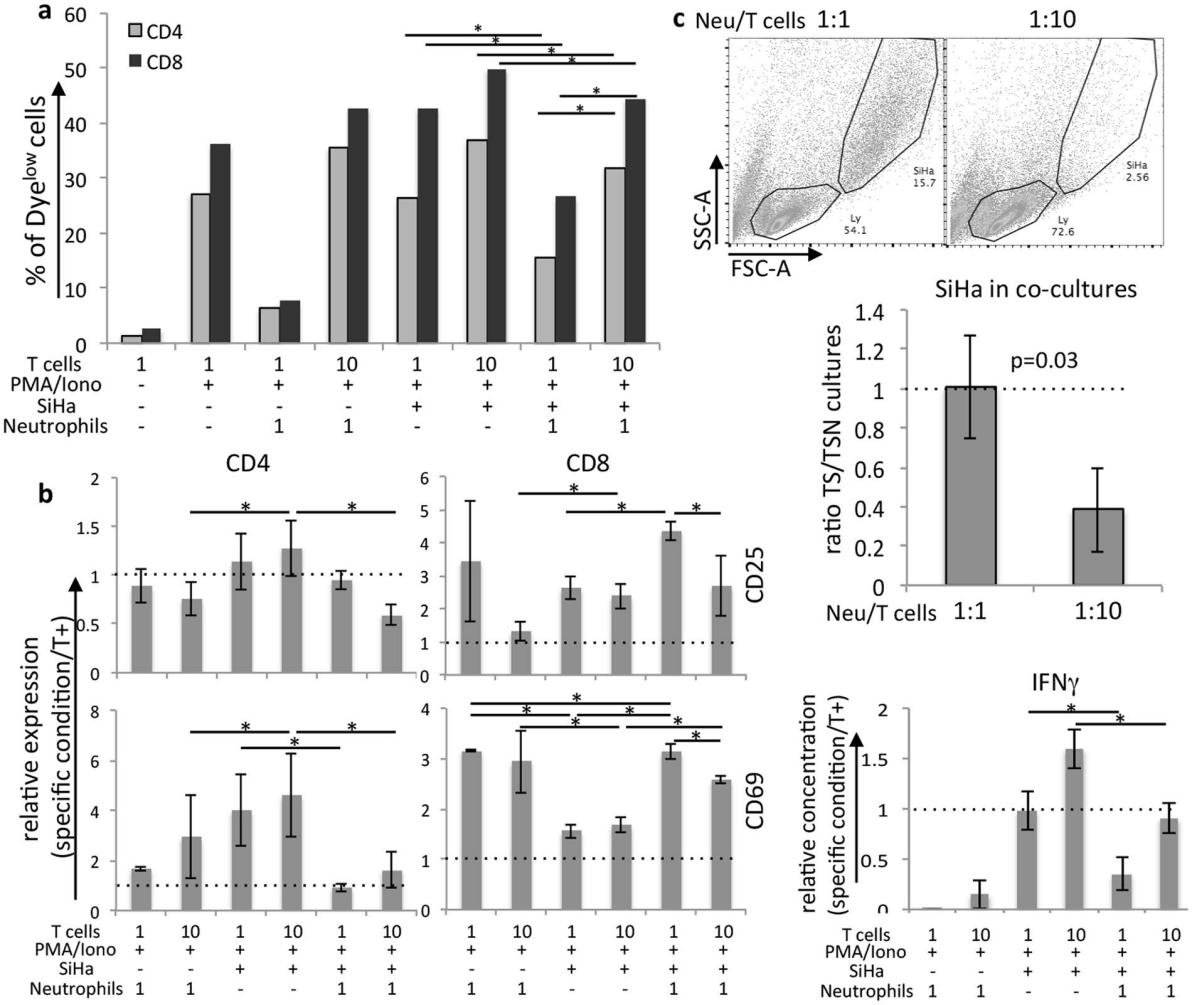
Effects of neutrophils over T cell activity in a tumor-like environment. Cell proliferation dye labeled T cells were stimulated with 100 ng/ml TPA and 1 μg/ml ionomycin (TPA/Iono) for 5 hours, before they were washed and added to neutrophils cultures (in 1 T cell: 1 neutrophil or 10 T cells: 1 neutrophil ratios), or 4 days SiHa spheroid cultures (SiHa) or neutrophils previously incubated for 5 hours with 4 days SiHa spheroid cultures. After 5 days incubation, cells were harvested and labeled with anti-CD45, CD4, CD8, CD25, CD69, fixed, and labeled with 10 μg/ml DAPI and then analyzed by flow cytometry. (**a**) For proliferation analysis, we gated out debris and doublets, than gated on CD45^+^ cells and then CD4^+^ or CD8^+^ cells, where we evaluated the percentage of cells that had low Cell Proliferation dye fluorescence, compared to a non-stimulated T cell control. Experiment representative of 4 independent ones (there was variation among cells from different donors). Numbers indicated in the graph, show the average of the proliferation ratios between T cells in 1:1 and 1:10 cultures with neutrophils incubated with SiHa spheroids in 4 independent experiments. (**b**) Relative CD25 and CD69 expression in the same cultures described in (**a**), as well as concentration of IFNγ in the cultures supernatant measured with the CBA kit (BD Biosciences). Results are the average of 4 independent experiments, and were tested by one-way ANOVA, either comparing 1:1 culture conditions and 1:10 culture conditions; ^*^indicates significant differences between experimental groups, dotted lines indicate ratio 1. (**c**) Depletion of SiHa cells from cultures with 10:1 T cells/neutrophils. Dot-plots on top represent the SiHa cells gate obtained after cells harvesting and flow cytometry analyzes. Total absolute cell numbers were determined by counting cells suspensions using a hemocytometer. These numbers were multiplied by the frequency of SiHa cells to obtain the absolute SiHa numbers per experimental condition. The graph bellow shows the ratio of SiHa cell numbers in SiHa/T cells cultures (TS) divided by the SiHa cell numbers in SiHa/neutrophils/ T cells cultures (TSN) in the different T cell/neutrophil experimental ratios. Results are the average of 3 independent experiments, tested by t-test, p value indicated in the graph, dotted lines indicate ratio 1.

In cancer, neutrophils have been shown to display both pro and anti-tumoral effects^20^. Inhibition of T cell activity is one of the mechanisms by which they may facilitate tumor growth. Our previous results indicated that neutrophils from cervical cancer might display a suppressive phenotype. To test this hypothesis, we used SiHa tumor spheroids to test the interaction between neutrophils and T cells in a tumor like microenvironment. In these experiments, CellTrace labeled T cells were stimulated *ex vivo*, washed and then added to a co-culture of neutrophils and SiHa spheroids. Four days later, we harvested the cells and labeled with antibodies for flow cytometry analysis. To mimic what we observed in the cervical biopsies, we set up cultures with 1:1 or 10:1 T cells/neutrophils ratios. Tumor cells did not inhibit T cell activity. On the contrary, T cells proliferated, expressed activation markers, secreted IFNγ and even eliminated tumor cells in the spheroids (Fig. 6a–c). However, when incubated with neutrophils, in 1:1 proportion, T cells were inhibited. We observed significant decrease in T cell proliferation, expression of CD69, mainly in CD4 T cells, and expression of CD25 in both CD4 and CD8 T cells (such low CD25 expression may be indicative of failure to proliferate upon mitogen stimulation). We also observed inhibition of IFNγ secretion. On the other hand, we observed maintenance of SiHa cells in the cultures (Fig. 6b,c). Therefore, although T cells were functional in the presence of tumor cells, they were inhibited by the addition of neutrophils. This effect was almost completely lost if we seeded 10 T cells to 1 neutrophil, indicating that in excess, T cells were resistant to neutrophils inhibitory effects. One can also observe that neutrophils alone could inhibit proliferation of T cells and IFNγ secretion, when in 1:1 culture ratio. However, this effect was probably mediated by the release of toxic molecules upon death of the neutrophils.

Our conclusion from these experiments was that in high frequency, neutrophils in the tumor microenvironment could inhibit T cells activity.

### Systemic effects were observed in cancer patients, where there was higher concentration of circulating G-CSF, CD66b^+^ immature neutrophils and lower antigen presenting potential

Our laboratory has previously shown, using experimental models, that HPV positive tumor cells expressed different cytokines *in vivo*, including G-CSF, IL-6 and IL-8^26, 27^. Others have shown that patients with cervical cancer have high circulating G-CSF concentration and leukocytosis^28^. G-CSF induces neutrophil ontogeny, mobilization from the bone marrow, recruitment and viability^29^. Moreover, G-CSF and IL-6 may promote expansion of myeloid derived suppressor cells, which can interfere with T cell activation and anti-tumor responses.

We wanted to study potential systemic effects of cervical lesions on the immune system of the patients, and also determine at which point in lesion progression we would be able to detect these alterations. We focused on parameters previously associated with worse prognosis in cervical cancer patients, as G-CSF concentration, or in cancer in general, as monocyte derived dendritic cells decreased antigen presentation potential.

**Figure 7:**
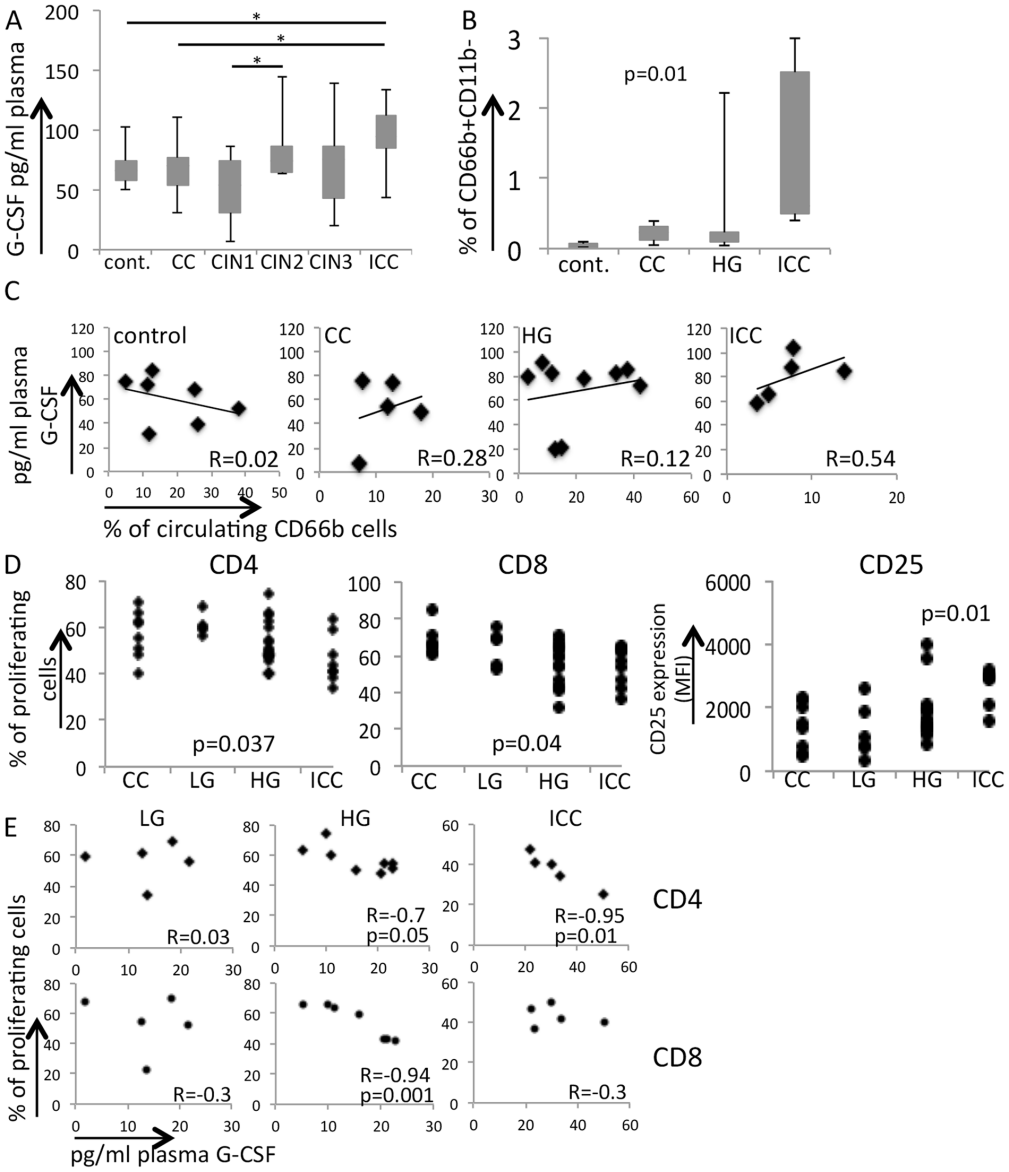
Cervical lesion systemic effects. (**A**) G-CSF plasma concentrations, measured by ELISA. We used plasma from 8 control donors, from 10 CC patients, 8 CIN1, 11 CIN2, 18 CIN3 and 17 ICC patients. Results were compared by one-way ANOVA, significant differences are indicated by *. (**B**) Frequency of immature circulating neutrophils. The mononuclear cells from the Ficoll-Paque gradient were harvested and labeled with anti-CD66b and CD11b. The graph shows the frequency of CD66b^+^CD11b^−^ cells in each experimental group, which contained 5 controls, 4 CC patients, 13 high grade lesion patients (HG) and 11 ICC patients. We had only 2 patients with CIN1 lesions, therefore, had to exclude this group from this analysis. Again, results were tested with one-way ANOVA, and the p value is indicated. (**C**) Correlation between G-CSF plasma concentration and frequency of circulating immature neutrophils CD66b^+^. We used Pearson correlation to compare these parameters, R values indicated in each graph. (**D**) Allogeneic T cell stimulation. Cell proliferation dye labeled T cells from control donors were incubated for 4 days with MoDCs from patients of the indicated groups. After incubation, cells were harvested, labeled with CD4, CD8 and CD25 and analyzed by flow cytometry. Cell proliferation data shows the frequency of cells within the CD4^+^ or CD8^+^ T populations that diluted the proliferation dye, therefore, that proliferated during the experiments. CD25 expression, represented as median fluorescence intensity (MFI) was gated on CD4 T cells. We used one-way ANOVA to compare experimental groups. In all cases, samples from ICC patients were significantly different from the other patients. CC – cervicitis, LG – cells from patients with low grade lesions (or CIN1), HG – cells from patients with high grade lesions (CIN2/3), ICC – cells from patients with invasive cervical carcinoma. (**E**) Pearson correlations between T cell proliferation determined in D and plasma G-CSF concentrations, determined in B; R and p values are indicated in the plots.

We observed that our ICC patients had the highest G-CSF circulating levels, although patients with CIN2 and CIN3 also had higher levels of circulating G-CSF concentration than patients with CIN1, cervicitis or controls with no reported cervical lesion (Fig. 7A). Therefore, our data indicated that G-CSF concentration increased with lesion grade and we could detect this event before other systemic events and alterations in the local microenvironment were noticeable, at least as far as we were able to measure.

We observed that cancer patients had higher numbers of circulating low density CD11b^−^CD66b^+^ cells, which correspond to immature neutrophils^30, 31^ (Fig. 7B). Correlation between plasma G-CSF concentration and frequency of circulating CD11b^−^CD66b^+^ cells from the same patients showed us that, while there was no correlation between these parameters in patients with cervicitis or high grade CIN, there was a moderate positive correlation between these two parameters in patients with cancer (Fig. 7C), indicating that this cytokine may have a role in the accumulation of these cells in the blood of cancer patients. Unfortunately, we did not have enough patients with samples to perform a correlation test with the low grade CIN group.

G-CSF can modulate dendritic cells differentiated from monocytes (MoDC), inducing a tolerogenic phenotype^29^. Allogeneic antigen presentation assays using MoDC from our patients and T lymphocytes from healthy donors showed us that monocytes from cancer patients originated tolerogenic dendritic cells, which inhibited T cell proliferation and induced CD25 expression, which high expression may indicate regulatory phenotype (Fig. 7D). Again, we could compare G-CSF plasma concentrations with T cell proliferation induced by MoDCs of some of our patients. We found that there was no correlation between these parameters in patients with low grade lesions. We found, however, a negative correlation between CD4 (R = −0.07, p < 0.05) and CD8 (R = −0.9, p = 0.02) proliferation induced by MoDCs and G-CSF plasma concentrations from patients with CIN3 lesions, and also negative correlation between CD4 (R = −0.9, p < 0.05) proliferation and G-CSF plasma concentrations from ICC patients (Fig. 7E).

Finally, we have investigated the frequency of myeloid (Lin^−^HLA-DR^+^CD11c^+^CD123^−^) and plasmacytoid (Lin^−^HLA-DR^+^CD11c^−^CD123^+^) circulating dendritic cells in our cohort. We have found no significant differences in the frequency of these populations in our groups, with averages of 51.8% (DP 6.1%) of CD11c^+^ and 7.7% (DP 1.8%) of CD123^+^ cells within the Lin^−^HLA-DR^+^ PBMC population.

## Discussion

This work aimed to characterize changes in the local microenvironment and systemic parameters that could point to escape mechanisms that could participate in cervical cancer progression. One of our questions was if it was possible to detect alterations in the various measured parameters in precursor lesions. Curiously, although we could observe an increase in the pan-leukocyte infiltration in cervical precursor lesions, specific population frequencies changed significantly only in cancer samples. However, we could observe significant increase in G-CSF plasma concentration in patients with high grade precursor lesions, indicating that in spite of imperceptible, precursor lesions did influence the immune system of the patients systemically.

T cells were the most abundant population in all our samples, followed by neutrophils and macrophages. Accordingly, Givan and collaborators found similar frequencies of these populations in the cervix of women undergoing hysterectomy^32^. The increase in leukocyte recruitment in cancer patients may be due to an increase in tumor cell numbers and changes in tumor cell secretion profile, which would result in higher concentrations of cytokines and chemokines, leading to increased recruitment. Changes in the microbiome also could lead to alterations in the mucosal leukocyte populations. Although we could not determine a correlation between reported clinical infection with other agents than HPV and frequency of inflammatory infiltrate in cervical biopsies, we are currently sequencing 16 S bacterial DNA from all our samples, hoping to get in depth information about microorganisms present in the cervix of our patients.

While T cells, neutrophils and M2 macrophages infiltration increased in cancer samples, the opposite was observed with M1 macrophages and NK cells, resulting in relatively lower frequency of the last two populations in cervical cancer biopsies in comparison to other cell populations. Interestingly these are two potentially cytotoxic populations, which could eliminate tumor cells. The relative decrease in these populations may be due to changes in adaptive immune responses in cancer patients: for instance, regulatory T cells can deprive NK cells of IL-2 and decrease their activation and proliferation^33^, and there may be less M1 macrophages differentiation due to the lack of Th1 responses. Indeed, our data shows that cancer patients had tolerogenic MoDCs, which could lead to reduced T cell responses, and alterations in the tumor microenvironment. Interestingly, although G-CSF is known for its role as a growth factor for neutrophils, it can also modulate the activity of MoDCs, inducing a tolerogenic phenotype^29^. Indeed, in patients with cancer or high grade lesions, there was an inverse correlation between G-CSF plasma concentrations and T cell activation by MoDCs, suggesting that even precursor lesions can modulate systemic immune responses.

Data in the literature indicate that macrophages, the third most abundant population in our samples, display a pro-tumoral effect in cervical cancer^14^. Our laboratory has previously demonstrated, using experimental models, that macrophages from HPV associated tumors can suppress T cell anti-tumor activity^34^. We have thoroughly tested macrophage and T cell frequencies correlations and found only weak negative correlations. It is possible that because macrophages are less abundant than neutrophils in the cervical lesions, the last may have predominant effects on T cells, which does not mean that we can rule out potential suppressive effects macrophages may also have on T cells. Another factor to consider is that M2 macrophages may promote other important events in the tumor microenvironment as angiogenesis and resistance to chemotherapy^10, 13, 14^. The increase in the frequency of the M2 population, therefore, may have different effects on cervical tumor progression, other than supression of immune responses.

Neutrophils were the second most abundant population in our samples. Among their effects, neutrophils together with tumor cells promoted an IL-6 and IL-8 rich environment. This effect may add to the inefficiency of T cell activation, as it has been described that these cytokines can promote accumulation of regulatory T cells in mesotheliomas^35^. Moreover, IL-6 together with PGE2 is known to promote tolerogenic phenotype in antigen presenting cells^14^, again inhibiting T cell activity. It is possible that our cancer patients have higher levels of IL-6 in the circulation, as been shown in other cancer patients. However, we have yet to test if this is correct.

All CIN lesions and cancer samples displayed a strong negative correlation between neutrophils and T cells. This pattern was not present in cervicitis samples. This data suggests that dysplastic or neoplastic cervical tissue controlled the microenvironment in a different manner than inflamed cervical mucosa. In the gynecological practice, cytology tests are performed periodically to detect cellular alterations in the cervix and avoid cervical cancer progression. In some cases, the results are atypical squamous cells (ASC-US, Atypical squamous cells of undetermined significance, and ASC-H, cannot exclude high grade lesion), which bring uncertainty to both the patient and physician, and require HPV testing and follow up to try to determine if there is a lesion present and which measures to take to avoid cancer progression. It will be interesting to test if it is possible to use the lymphocyte/neutrophil ratio in cytology tests to differentiate CIN lesions from other atypical cervical abnormalities.

Tumor associated neutrophils (TAN) may suppress T cell responses^36^. Using an *in vitro* model, we could show that tumor cells modulated neutrophils incubated with SiHa spheroids, which in turn suppressed T cell proliferation and activity. How T cells could be eliminating the SiHa cells, we do not know at this point, probably through secretion of cytotoxic factors. There is evidence that neutrophils exposed to G-CSF may acquire a tolerogenic phenotype, through IL-10 secretion^37^. This seems not be the case in our system, since we found no IL-10 secretion in neutrophil/tumor cells cultures. Other mechanisms by which neutrophils could inhibit T cells are production of ROS, expression other cytokines as IL-6, or PD-1L expression^20, 38^. However, we still need to investigate the interaction between tumor cells, neutrophils and T cells to understand the mechanisms behind our observations.

Circulating NLR has been used as prognostic marker for different types of cancer, including cervical cancer^16^. In our study, we found higher frequency of low density CD66b^+^ cells in the blood, which could eventually infiltrate the cervix of our patients. Although we have not addressed this issue specifically, as our data is represented in percentage of cells, the increase of this population definitely indicates a higher CD66b^+^/lymphocyte ratio in our cancer patients. Interestingly, recent data has shown that a relatively high percentage of cervical cancer patients have leukocytosis (14%) and high circulating G-CSF levels, which are poor prognostic markers^15, 39, 40^. As Mabuchi and collaborators observed a correlation between G-CSF concentration and leukocytosis, we identified a positive, although moderate, correlation between G-CSF serum concentration and frequency of low density CD66b^+^ cells in the blood of cervical cancer patients.

G-CSF signals are transduced through the G-CSFR and different signaling pathways, including STAT3 (Signal transducer and activator of transcription 3)^41^. Chronic STAT3 activation has been correlated with tolerance and cancer progression^41^. Therefore, it is possible that the G-CSF/STAT3 axis is controlling part of the systemic and local effects we observed in this study. Locally, we can hypothesize that other factors, as IL-6, IL-8 and IL-10, act together with G-CSF to influence immune responses. Koshiol and collaborators have shown that women positive for cervical HPV infection have higher local G-CSF concentration^42^, supporting our findings. However, it is possible that other cytokines secreted in the lesion microenvironment, as IL-6 and IL-10, and that also signal through STAT3 may have local and systemic effects, as well, also promoting tolerance. Therefore, it is likely that other cytokines and inflammatory factors may also play a role in the control of leukocyte production, recruitment and activation. There is actually evidence for the role of IL-10 and IL-6 in promoting tolerance toward HPV antigens^14, 26^.

Our results indicate that as lesions progress from low to high grade to cancer, there is an increase in cytokine production, for instance G-CSF, which causes systemic effects that influence the triggering of adaptive immune responses, partially inhibiting antigen presentation and therefore T cell activation. Locally these effects cooperate with the accumulation of T cells, neutrophils and M2 macrophages, but decrease of NK cells and M1 macrophages, reflecting a less cytotoxic environment, which again circles back to the diminished T cell activation. As far as we now, this is the first comprehensive analyzes of local and systemic effects of cervical lesions on the immune system, which may be of importance, not only for addressing cervical cancer treatment, but also may help with abnormal cervical cytology diagnosis.

## Materials and Methods

### Study design

This study was approved by the Institute of Biomedical Sciences Ethics Committee, process 1061 and Ethics Committee for research project analyzes of the Hospital das Clínicas, School of Medicine, Universidade de São Paulo (National Committee in Research Ethics project 03375412.4.0000.5467). All patients and healthy donors signed an informed consent formulary before sample harvesting. All methods followed the guidelines established by CONEP, Brazilian National Committee in Research Ethics.

Patients with positive cytology results were referred to the Gynecology ambulatory service of the Hospital das Clínicas, School of Medicine, Universidade de São Paulo, Brazil. Two to six months passed between the cytology and the exam at the Hospital das Clínicas, so that some of the patients, although still HPV positive, had already spontaneously eliminated their lesions. In the Gynecology service, patients were submitted to colposcopic examination. At the physician discretion, patients were submitted to an excisional procedure or biopsy harvesting for diagnostic. In both cases, a second biopsy within the lesion area was taken for our study and immediately placed in a tube containing 5 ml of sterile RPMI1640 (LifeTechnologies, Carlsbad, CA). Patients also donated 10 ml of peripheral blood. Patients pregnant or with any type of immunosuppression were excluded from study. The study was designed to compare samples from 20 patients from each of the groups: cervicitis, CIN1, CIN2, CIN3 and invasive cancer. However, after histopathological analyses, some of the samples were re-classified, which led to a variation in the number of samples per group. We enrolled a total of 143 patients, but we able to analyze a total of 81 biopsies (Fig. 1). Most of the discarded biopsies had insufficient number of cells or low viability after tissue digestion; other had insufficient clinical information.

For some experiments, we needed blood cells from healthy donors. We harvested blood from 20 controls, with ages ranging from 25 to 50 years old, all with the same exclusion criteria than our patients, and no reported cancer. Blood samples were collected in Heparin Vacutainer tubes (Becton-Dickinson, Sunnyvale, CA). Samples were processed within 3 to 4 hours after harvesting.

### Tissue processing

Cervical biopsies were split in two fragments. The smaller was used for DNA extraction and HPV genotyping, the largest one was finely minced and digested with 1 mg/ml Collagenase I and IV in MTH (1x Hanks’ buffered salt solution with 15 mM HEPES pH 7.4, 5% Fetal Bovine Serum, FBS, 0.5 U/ml DNAse I) at 37 °C, 1300 rpm using a Thermomixer (Eppendorf, New York, NY) for approximately 40 minutes. Cells were then filtered, washed in MTH and counted with Trypan blue to determine cell number and viability (usually between 90% and 95%). Contaminant enzymes may digest cells surface markers. The use of FBS in the digestion buffer inhibits the potential contaminants, avoiding non-specific digestion^43^.

Peripheral blood monocytic cells (PBMCs) were isolated from peripheral blood through density gradient centrifugation in Ficoll-Paque (GE Life Sciences, Marlborough, MA). Plasma samples were frozen in aliquots for later use.

Neutrophils were isolated from peripheral blood, starting with 2 ml of blood, from which we recovered 1.4 ± 0.28 million CD15^+^^44^.

### Flow cytometry

Cervical cell suspensions were labeled with directly conjugated monoclonal antibodies (Supplementary Information 2). All antibodies were tittered to optimal concentration before use. Unstained cells were used to define gates for positive labeled. For staining, cells were blocked with 1:100 FcBlock (ThermoFisher, Waltham, MA) for 15 min at 4 °C, prior to addition of antibodies and incubation for 20 min at 4 °C. Cells were then washed and fixed in buffered 2% paraformaldehyde. All cells were analyzed by flow cytometry, where at least 5,000 events of the CD45^+^ population were acquired.

The gating strategy for biopsy analyses was the following: exclusion of debris and doublets, gate on CD45 X FSC to identify the leukocyte population, and within this population, we plotted CD45 × SSC to identify 3 CD45^+^ populations, with high, intermediate and low SSC. Within each of these gates, we analyzed the frequency of different populations, using specific antibody cocktails as shown in Supplementary Information 1a,b.

Flow cytometry of blood cells or cell cultures were performed with gating on FCS × SSC for debris exclusion, followed by doublet exclusion, and then analyses of the different markers, including cell proliferation dye.

All flow cytometry was acquired in a FACSCanto II (BD Biosciences, San Jose, CA), and data was analyzed using the FlowJo software 7.6.5 v (TreeStar, Ashland, OR).

### T cell and neutrophil *in vitro* assays

All cell culture incubations were done in 10% FBS in RPMI1640, at 37 °C in a 5% CO_2_ atmosphere.

#### MoDC differentiation

monocytes were isolated from PBMCs by adhesion to cell culture plates for 12 hours. Cells were treated with 50 ng/ml GM-CSF and 50 ng/ml IL-4 (both from PeproTech Inc., Mexico) for 5 days, followed by a 48 hours treatment with 50 ng/ml TNFα (PeproTech Inc., Mexico) to induce differentiation and activation of MoDCs. In the 7^th^ culture day, cells were harvested and counted. An aliquot was taken from a few random samples to confirm the DC phenotype (CD11c^+^CD14^−^CD86^+^HLA-DR^+^) (Supplementary Information 1c). The other cells were used for mixed leukocyte reactions.

#### Mixed leukocyte reaction

MoDCs were incubated with previously labeled T cells (Cell Proliferation Dye eFluor 450, ThermoFisher, Waltham, MA), in a proportion of 1 DC to 10 T cells (1 × 10^5^ T cells per well) in triplicates. T cells were isolated from PBMCs from healthy donors using the Pan T cell isolation kit according to the manufacturer’s protocol (Miltenyi Biotec, Germany). After 5 days, cells were harvested, labeled with anti-CD3, anti-CD4 and anti-CD8 antibodies and analyzed by flow cytometry, where at least 10^4^ events were acquired per sample.

#### SiHa spheroids

5 × 10^3^ SiHa cells were seeded on 1% agarose in U bottom plates and incubated for 4 days.

#### Tumor cell effects on neutrophils

3 × 10^4^ circulating neutrophils from healthy donors were seeded on 1% agarose in U bottom plates or on 4 days SiHa or HeLa spheroids. At 4, 8 and 24 hours of incubation, we harvested cells to count, label with anti-CD16, anti-CD66b and DAPI^45^, and analyze by flow cytometry, where 3 × 10^4^ events were acquired. Supernatants were used for cytokine detection using the Human Inflammatory Cytokine Bead Array Kit (BD Biosciences, Carlsbad, CA).

#### Interaction between neutrophils and T cells in tumor spheroids

Circulating T cells and neutrophils were isolated from healthy donors as previously described. Cell Proliferation Dye labeled T cells were stimulated with 1 μg/ml ionomycin (SigmaAldrich, St Louis, MO) and 100 ng/ml TPA (or PMA) (Cell Signaling Technology, Danvers, MA) for 5 hours, then washed in fresh medium and added to 4 days SiHa spheroids, or neutrophils, or neutrophils and incubated with 4 days SiHa spheroids for 5 hours. To mimic the *in vivo* situation, we added 1 T cell for each neutrophil (3 × 10^4^ each) or 10 T cells for each neutrophil in culture. Five days later, cells were harvested and labeled with anti-CD4, anti-CD8, anti-CD11b, anti-CD69 and anti-CD25 and analyzed by flow cytometry.

#### ROS production by neutrophils

3 × 10^4^ neutrophils isolated from blood samples or biopsies, the last one using positive selection with anti-CD15 beads and MS columns (Miltenyi Biotec, Germany) were treated with 100 ng/ml TPA (Cell Signaling Technology, Danvers, MA) for 15 min in culture, and then incubated with 2 μl of Dihydrorhodamine 123, DHR, (ThermoFisher, Waltham, MA) for another 15 min. Cells were immediately washed, labeled with anti-CD11b, anti-CD66b and anti-CD45, and analyzed by flow cytometry, where a minimum of 5 × 10^3^ events were acquired. As controls, we used non-stimulated neutrophils, and neutrophils not labeled with DHR.

### HPV genotyping

150 ng of phenol/chloroform extracted DNA from cervical biopsies were used for PCR amplification using PGMY09/11 generic primers^46^. The amplification products were tested through the Roche Linear Array HPV genotyping test (Roche Molecular Diagnostics, Alameda, CA) capable of detecting 37 different HPV types.

### G-CSF plasma concentration

G-CSF plasma concentrations were determined by ELISA (ThermoFisher, Waltham, MA), according to the manufacture’s instructions.

### Histology

4 μm sections from paraffin embedded tissues were processed and stained with HE at the Pathology Department of the School of Medicine, Universidade de São Paulo. Images were acquired using an Olympus BX61 microscope with attached Olympus camera, and accompanying software for image acquisition. Images were not submitted to edition or modification.

### Statistical analyses

Results involving comparing more than two experimental groups were tested by one-way analysis of variance (ANOVA), followed by Bonferroni pair comparison. Testing between 2 experimental groups was performed by t-test or Mann-Whitney test according to normal distribution assumption. Correlation between populations or biological parameters was determined by Pearson correlation. Level of significance accepted was 5%. All tests were conducted using the SPSS statistical package for Windows v1.8 or Microsoft Excel.

## Electronic supplementary material

Supplementary Information 
