# Supplementary material for: Local and systemic immunomodulatory mechanisms triggered by Human Papillomavirus transformed cells: a potential role for G-CSF and neutrophils
Source: Sci Rep. 2017 Aug 21;7:9002. doi: 10.1038/s41598-017-09079-3 (PMC5566396; doi:10.1038/s41598-017-09079-3)
Supplement: Supplementary file 1 — Supplementary Information [file 41598_2017_9079_MOESM1_ESM.pdf]

SUPPLEMENTARY INFORMATION

Local and systemic immunomodulatory mechanisms triggered by Human Papillomavirus transformed cells: a potential role for G-CSF and neutrophils.

Karla Lucia Fernandez Alvarez, Mariana Beldi, Fabiane Sarmanho, Renata Ariza Marques Rossetti, Caio Raony Farina Silveira, Giana Rabello Mota, Maria Antonieta Andreoli, Eliana Dias de Carvalho Caruso, Marcia Ferreira Kamillos, Ana Marta Souza, Haydee Mastrocalla, Maria Alejandra Clavijo-Salomon, José Alexandre Marzagão Barbuto, Noely Paula Lorenzi , Adhemar Longatto-Filho, Edmund Baracat, Rossana Verónica Mendoza Lopez, Luisa Lina Villa, Maricy Tacla, Ana Paula Lepique[*].

Supplementary Information 1. Gating strategy for analyses of the leukocyte infiltrate in cervical biopsies (a,b) and MoDC phenotype (c).

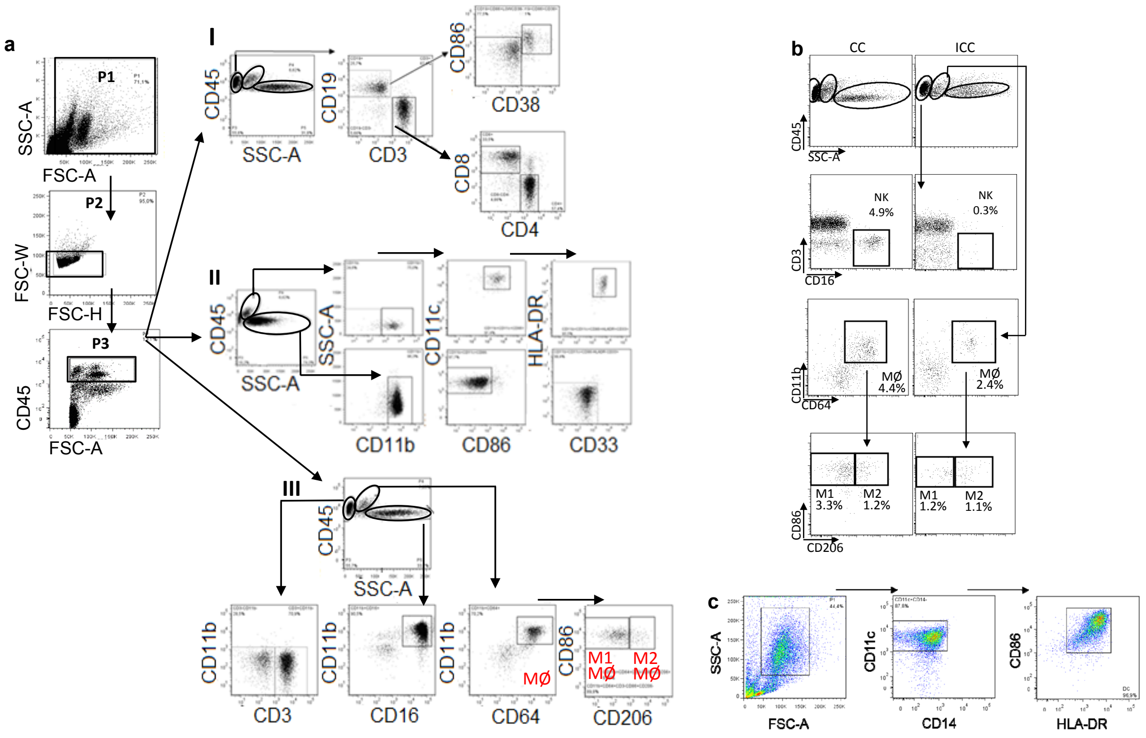

a. Gating strategy for cervical biopsies. P1, P2 and P3, were sequentially gated to exclude debris and doublets. In P3, the CD45+ populations were identified. Then CD45 was plotted against SSC to identify populations with lymphoid, macrophage or granulocytic characteristics, low, intermediate and high SSC, respectively. All populations were tested for all markers. However, in this figure we are indicating only the positive populations leading to the final phenotype of each one. The panels I, II and III correspond to different sets of antibodies that were applied according to the number of cells in each biopsy. Biopsies with more than $3x10^5$ cells were incubated with the 3 antibody sets (I, II and III). Otherwise, our priority was the II and III sets of antibodies. Therefore, not all populations were characterized in all biopsies. **b.** Example of gating for identification of NK cells and of NK cells and

macrophages frequencies in a patient with cervicitis, CC, and invasive carcinoma, ICC. **c.** Phenotype of MoDCs, after differentiation with 50ng/ml IL-4 and 50 ng/ml GM-CSF (5 days) and maturation with 50 ng/ml TNFα (48 hours), by flow cytometry. The population with high FSC and high SSC was positive for CD11c, negative for CD14, positive for CD86 and HLA-DR.

Supplementary Information2.

Antibodies used in the study and phenotype of leukocyte main populations in the cervix.

Antibody	clone	conjugate	neu	CD4	CD8	NK	M1	M2	B cells
CD3	UCHT1	V450 or APC	-	+	+	-	-	-	-
CD4	RPA-T4	APC-Cy7 or APC	-	+	-	-	-	-	-
CD8	HIT8a	PE or FITC	-	-	-	-	-	-	-
CD11b	ICRF44	APC	+	-	_	-	+	+	-
CD11c	B-ly6	PECy5 or FITC	-	nd	nd	nd	+	+	nd
CD14	M5E2	APC	-	nd	nd	nd	nd	nd	nd
CD15	HI98	PECy7	+	nd	nd	nd	nd	nd	nd
CD16	3G8	Biotin or PE	+	-	_	+	-	-	-
CD19	1D3	APC-Cy7 or APC or PECy5	-	-	_	-	-	-	+
CD25	M-A251	PECy5	nd	nd	nd	nd	nd	nd	nd
CD33	HIM3-4	FITC	-	nd	nd	nd	+	+	nd
CD45	H130	BV510	+	+	+	+	+	+	+
CD62L	DREG-56	V450	+	nd	nd	nd	nd	nd	nd
CD64	10.1	PerCP-Cy5.5	nd	nd	nd	nd	+	+	
CD66b	G10F5	PE	+	nd	nd	nd	nd	nd	nd

CD69	FN50	PECy7	nd	nd	nd	nd	nd	nd	nd
CD80	L3007.4	PE or Alexa 700	-	-	_	-	+	+	+/-
CD86	2331 (FUN-1)	PE-Cy7	-	-	_	-	+	+	+/-
CD123	9F5	BV421	nd	nd	nd	nd	nd	nd	nd
CD206	19.2	FITC	-	nd	nd	nd	-	+	nd
HLA-DR	G46-6	APC-H7 or FITC	-	nd	nd	nd	+	+	+
			SSC high	SSC low	SSC low	SSC low	SSC int.	SSC int.	SSC low

All antibodies were purchased from BD Biosciences (Carlsbad, CA).
